# Integrative analysis of expression profile indicates the ECM receptor and LTP dysfunction in the glioma-related epilepsy

**DOI:** 10.1186/s12864-022-08665-8

**Published:** 2022-06-08

**Authors:** Zhi-Bin Wang, Jian Qu, Pan Xie, Zhi-Quan Yang, Chen-Xue Mao, Ying Zhang, Zheng-Wen He, Zhuan-Yi Yang, Xiao-Yuan Mao, Zhao-Qian Liu

**Affiliations:** 1 Department of Clinical Pharmacology, Hunan Key Laboratory of Pharmacogenetics, and National Clinical Research Center for Geriatric Disorders, Xiangya Hospital, Central South University, Changsha, 410008 P. R. China; 2 Institute of Clinical Pharmacology, Engineering Research Center for applied Technology of Pharmacogenomics of Ministry of Education, Central South University, Changsha, 410078 P. R. China; 3 Department of Pharmacy, The Second Xiangya Hospital, Central South University, Changsha, 410011 P. R. China; 4 Departments of Neurosurgery, Xiangya Hospital, Central South University, Changsha, 410008 P. R. China; 5 Departments of Pharmacy, Xiangya Hospital, Central South University, Changsha, 410008 P. R. China; 6 Department of Neurosurgery, Hunan Cancer Hospital and the Affiliated Cancer Hospital of Xiangya School of Medicine, Central South University, Changsha, 410013 P. R. China

**Keywords:** Glioma-related epilepsy, lncRNAs, mRNAs, miRNAs, LTP, ECM receptor

## Abstract

**Background:**

Seizures are a common symptom in glioma patients, and they can cause brain dysfunction. However, the mechanism by which glioma-related epilepsy (GRE) causes alterations in brain networks remains elusive.

**Objective:**

To investigate the potential pathogenic mechanism of GRE by analyzing the dynamic expression profiles of microRNA/ mRNA/ lncRNA in brain tissues of glioma patients.

**Methods:**

Brain tissues of 16 patients with GRE and 9 patients with glioma without epilepsy (GNE) were collected. The total RNA was dephosphorylated, labeled, and hybridized to the Agilent Human miRNA Microarray, Release 19.0, 8 × 60 K. The cDNA was labeled and hybridized to the Agilent LncRNA + mRNA Human Gene Expression Microarray V3.0, 4 × 180 K. The raw data was extracted from hybridized images using Agilent Feature Extraction, and quantile normalization was performed using the Agilent GeneSpring. P-value < 0.05 and absolute fold change > 2 were considered the threshold of differential expression data. Data analyses were performed using R and Bioconductor.

**Results:**

We found that 3 differentially expressed miRNAs (miR-10a-5p, miR-10b-5p, miR-629-3p), 6 differentially expressed lncRNAs (TTN-AS1, LINC00641, SNHG14, LINC00894, SNHG1, OIP5-AS1), and 49 differentially expressed mRNAs play a vitally critical role in developing GRE. The expression of GABARAPL1, GRAMD1B, and IQSEC3 were validated more than twofold higher in the GRE group than in the GNE group in the validation cohort. Pathways including ECM receptor interaction and long-term potentiation (LTP) may contribute to the disease’s progression. Meanwhile, We built a lncRNA-microRNA-Gene regulatory network with structural and functional significance.

**Conclusion:**

These findings can offer a fresh perspective on GRE-induced brain network changes.

**Supplementary Information:**

The online version contains supplementary material available at 10.1186/s12864-022-08665-8.

## Introduction

Glioma is the most common type of primary brain tumor, and the vast majority of glioma patients suffer from seizures. The concept of glioma-related epilepsy (GRE) was firstly mentioned by Hughlings Jackson in 1882. Gliomas and seizures may share comparable molecular pathways, and glioma-induced structural alterations may lead to epilepsy [1]. In a study of 406 individuals with glioma, 31% of patients experienced seizures during their illness, with the majority (72%) having progressive conditions [2]. Despite the benefits of anti-seizure drugs, chemotherapy, and radiation therapy for seizure management, overall seizure control for GRE remains inadequate [3]. Given that seizures cause a serious effect on the glioma patients, research in this area is crucial.

Regarding the pathogenesis of GRE, there are currently many different theories. Pallud et al. pointed out that glutamatergic and γ-aminobutyric acid (GABA) ergic alterations in gliomas may lead to epileptogenesis [4]. Since gliomas employ the neurotransmitter glutamate as a “tumor growth factor” to promote glioma cell proliferation and invasion, glutamate homeostasis is disrupted, resulting in higher extracellular glutamate concentrations. GABAergic signaling, on the other hand, is implicated in tumor development as well as paradoxical excitatory consequences. Wang et al. found that TSP2 overexpression in tumor tissue caused an increase in spine density and excitatory synapses in the peritumoral region, resulting in hyperexcitability in the peritumoral cortical networks [5]. Glioma-related epileptogenic mechanisms are multifaceted and intertwined, and they share similar mechanisms with glioma development processes and epileptogenesis mechanisms in other brain pathologies [6]. The precise mechanisms of GRE, on the other hand, are complex and unknown. Because they could not stratify patients into high- or low-risk seizure groups, early trials failed to establish an impact of the general administration of anti-epileptic medications for GRE patients.

MiRNA and lncRNA are epigenetic factors that control gene expression and play a role in neuron development, metabolism, and other activities [7–11]. Several miRNAs have been identified with GRE, including miR-128 and miRNA-196b [12, 13]. The regulation of lncRNAs is complicated and intertwined with mRNA, miRNA, and proteins. However, there are no publications on the relationship between lncRNAs and GRE. Integrative analysis of the regulatory networks of miRNAs, lncRNAs, and mRNAs to GRE is also scarce.

To date, there are a variety of mysteries on the GRE research as shown below: whether current treatments or responses are predictable, whether there are preventive strategies that protect glioma patients from seizures they may experience, and whether there are types of oncology treatments that might simultaneously alleviate seizures.

To make a comprehensive analysis of GRE-regulated transcriptional networks, we explored the dynamic expression profiles of lncRNA, mRNA, and miRNA in GRE and glioma without epilepsy (GNE) brain regions and the disrupted biological activities to discover the pathogenic causes of GRE. Finally, the RNA-RNA interactions were used to create a lncRNA-miRNA-mRNA ceRNA network.

## Materials and methods

### Sample collection

The study was approved by the ethics committee of Xiangya Hospital of Central South University (ethics approval number: CTXY-1300041–3). Brain tissues of 25 glioma patients for microarray study and 22 glioma patients for validation study with or without epilepsy were retrospectively obtained from the Affiliated Cancer Hospital of Xiangya School of Medicine (Changsha, Hunan, China) and Xiangya Hospital with informed consent. All of the glioma tissue was taken from the cerebral cortex. Tissues were obtained and kept at –80 °C until microarray analysis.

### RNA isolation and expression profiling

Total RNA was extracted with Invitrogen’s TRIzol Reagent, purified with NucleoSpin RNA clean-up, and quantified with a NanoDrop. Gel electrophoresis was used to check the RNA’s purity and integrity. MirVana miRNA Isolation Kit was used to purify total microRNA. Human miRNA Microarray, Release 19.0, 8 × 60 K (Agilent), which comprises 2006 human miRNAs, was used to profile microRNA expression. LncRNA + mRNA Human Gene Expression Microarray V3.0, 4 × 180 K, containing 34,235 human mRNAs and 34,808 human lncRNAs, was used to evaluate mRNA and lncRNA expression. Following the manufacturer’s instructions, 200 ng of total RNA was labeled and hybridized with the miRNA Complete Labeling and Hyb Kit (Agilent). Agilent G2565CA Microarray Scanner was used to scan the slides, and Agilent Feature Extraction Software v10.7 was used to analyze the scanned pictures of the microarray.

### Statistical analysis

The original data was normalized and log 2-scale transformed by GeneSpring GX software. Probes with a detection rate of less than 60% were filtered out. After pretreatment, we obtained 634 miRNA, 27,851 mRNAs, and 23,856 lncRNAs for subsequent analysis.

Comparisons between two groups were performed using the two-sided unpaired Student’s t-test. The false discovery rate was calculated using the Benjamini and Hochberg method. The cut-off values for statistical significance were adjusted *P*-value < 0.05 and fold change ≥ 2.

### Functional analysis of Differentially Expressed mRNAs (DEGs), miRNAs (DEMs), and lncRNAs (DELs)

The miRWalk database (integration of TargetScan, miRDB, and miRTarBase) [14] was used to predict DEM target mRNAs. DEM target lncRNAs were also investigated utilizing the ENCORI and LncBase v3 database [15, 16]. The pairwise combinations were filtered with a correlation coefficient less than -0.5.

Using the R package clusterProfiler [17], Gene Ontology (GO) analysis was performed on DEGs and the intersection of DEGs and mRNA targets of DEMs to derive GO terms. Gene lists were analyzed using the Kyoto Encyclopedia of Genes and Genomes (KEGG) database to discover probable GRE pathogenic pathways [18–20]. Gene Set Enrichment Analysis (GSEA) was performed using all mRNAs’ fold change value (FC).

The strategy “Guilt by association” was used to predict the prospective roles of each important hub DEM or DELs [21, 22]. The Pearson’s correlation coefficients for pairwise combinations between a DEM (or DEL) and all mRNAs were used to create a correlation matrix. GSEA was then used to calculate functional connections between DEM (or DEL) and KEGG terms.

### The lncRNA-miRNA-mRNA network construction

**Fig. 1:**
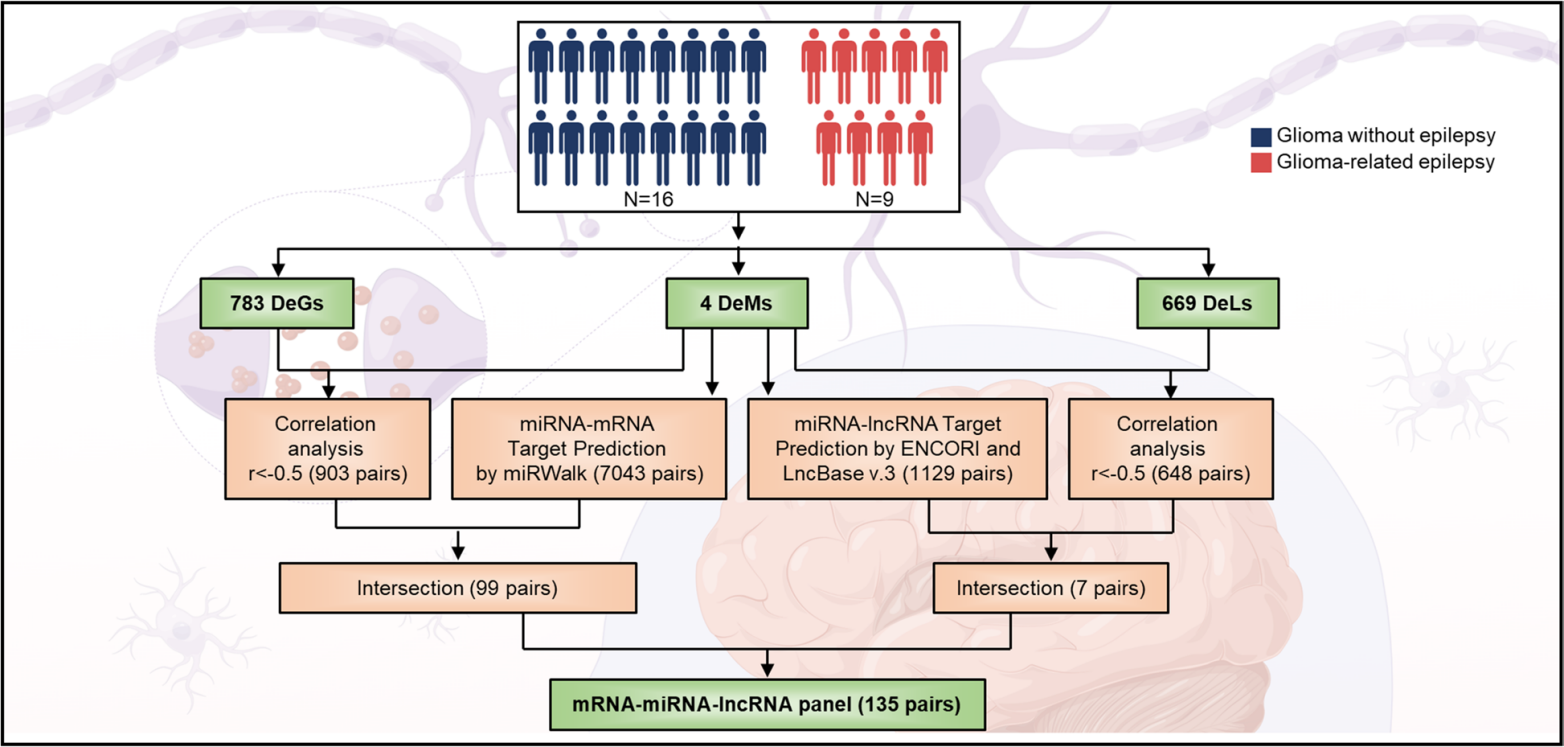
Flowchart summarizing the study design and methods used for lncRNA–miRNA–mRNA network construction. DEGs, differential expressed mRNAs; DEMs, differential expressed miRNAs; DELs, differential expressed long non-coding RNAs. (Drawing by Figdraw www.figdraw.com)

MiRNA-mRNA regulatory correlations and miRNA-lncRNA interactions were used to build the lncRNA-miRNA-mRNA network. The DEMs and their negatively regulated DEGs and DELs were chosen to create the lncRNA-miRNA-mRNA ceRNA networks. Using the Cytoscape software (v3.9.0), the lncRNA-miRNA-mRNA ceRNA network was displayed. The flowchart in Fig. 1 summarized the study strategy and methods for constructing the lncRNA-miRNA-mRNA network.

## Results

### Clinical characteristics of glioma patients with or without epilepsy

We analyzed 16 glioma patients without epilepsy and 9 glioma patients with epilepsy. For their medical intractability, all patients were referred to the Affiliated Cancer Hospital of Xiangya School of Medicine. At the time of operation, the average age of patients was 49 years old, ranging from 32 to 69. The demographic characteristics of patients selected in our present work was summarized in Table 1.

**Table 1:** Clinical data of glioma patients with or without epilepsy

Characteristic	Classes	GNE^a^	GRE^b^
Gender	Male	8	8
	Female	8	1
Age	Age ≤ 50	8	8
	Age > 50	8	1
Epilepsy	YES	0	9
	NO	16	0
Tumor grade	I-III	10	9
	IV	6	0
Site	Temporal lobe	5	3
	Frontal lobe	5	6
	Parietal lobe	5	0
	Occipital lobe	1	0

^a^glioma without epilepsy

^b^glioma-related epilepsy

All gliomas were primary without radiotherapy or chemotherapy was received before surgery. The type of epilepsy in GRE was sustained epilepsy

### Functional analysis of DEGs in GRE

**Fig. 2:**
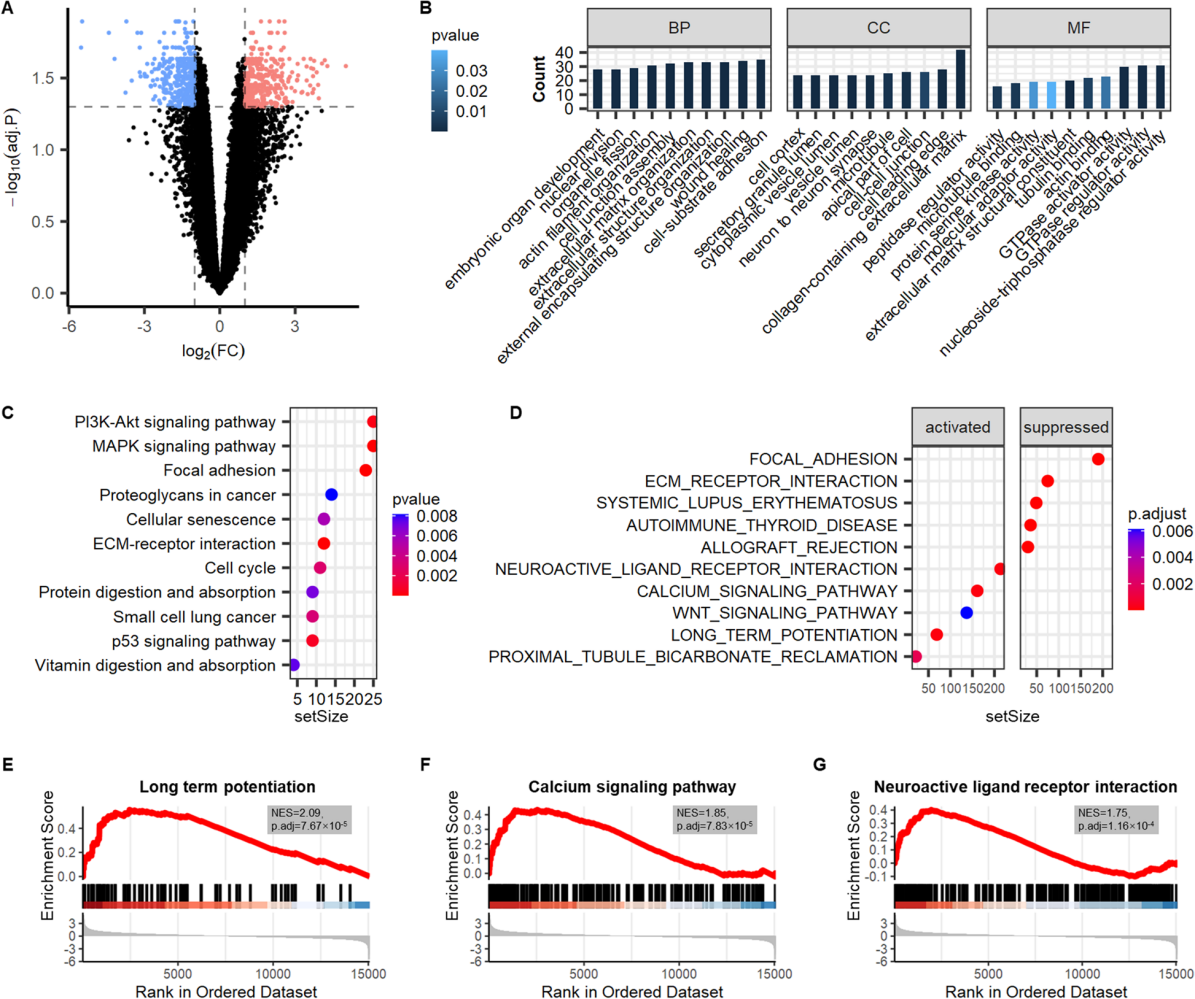
Identification and functional analysis of differentially expressed mRNAs (DEGs) in glioma-related epilepsy (GRE). **A** Volcano plots of DEGs. **B** Gene ontology (GO) annotations of DEGs. **C** KEGG pathway enrichment for DEGs analyzed by clusterProfiler package. **D** Gene set enrichment analysis (GSEA) terms were ordered according to their counts. FC, fold change; BP, biological process; CC, cellular component; MF, molecular function; NES, normalized enrichment scores. **E–G** Neuroscience related GSEA terms of GRE

The total 34,235 mRNAs were identified in glioma tissues. Between the two groups, 783 DEGs were discovered using the screening strategy for DEGs. When comparing GRE to GNE, 406 were up-regulated, and 377 were down-regulated (Fig. 2A). The biological roles of these genes were evaluated using GO and KEGG pathway analysis. DEGs were significantly enriched in GO terms, including cell-substrate adhesion, collagen-containing extracellular matrix, and nucleoside-triphosphatase regulator activity (Fig. 2B). DEGs were strongly enriched in KEGG pathways such as focal adhesion, MAPK signaling pathway, and ECM-receptor interaction (Fig. 2C). The GSEA analysis was performed to observe macroscopic pathways change for all mRNAs in GRE. Ten GSEA terms, including long-term potentiation (NES = 2.09, p.adj = 7.67 × 10^–5^), calcium signaling pathway (NES = 1.85, p.adj = 7.83 × 10^–5^), neuroactive ligand receptor interaction (NES = 1.75, p.adj = 1.16 × 10^–4^) were significantly enriched (Fig. 2D-G). They were all activated in GRE.

### Functional analysis of DEMs in GRE

**Fig. 3:**
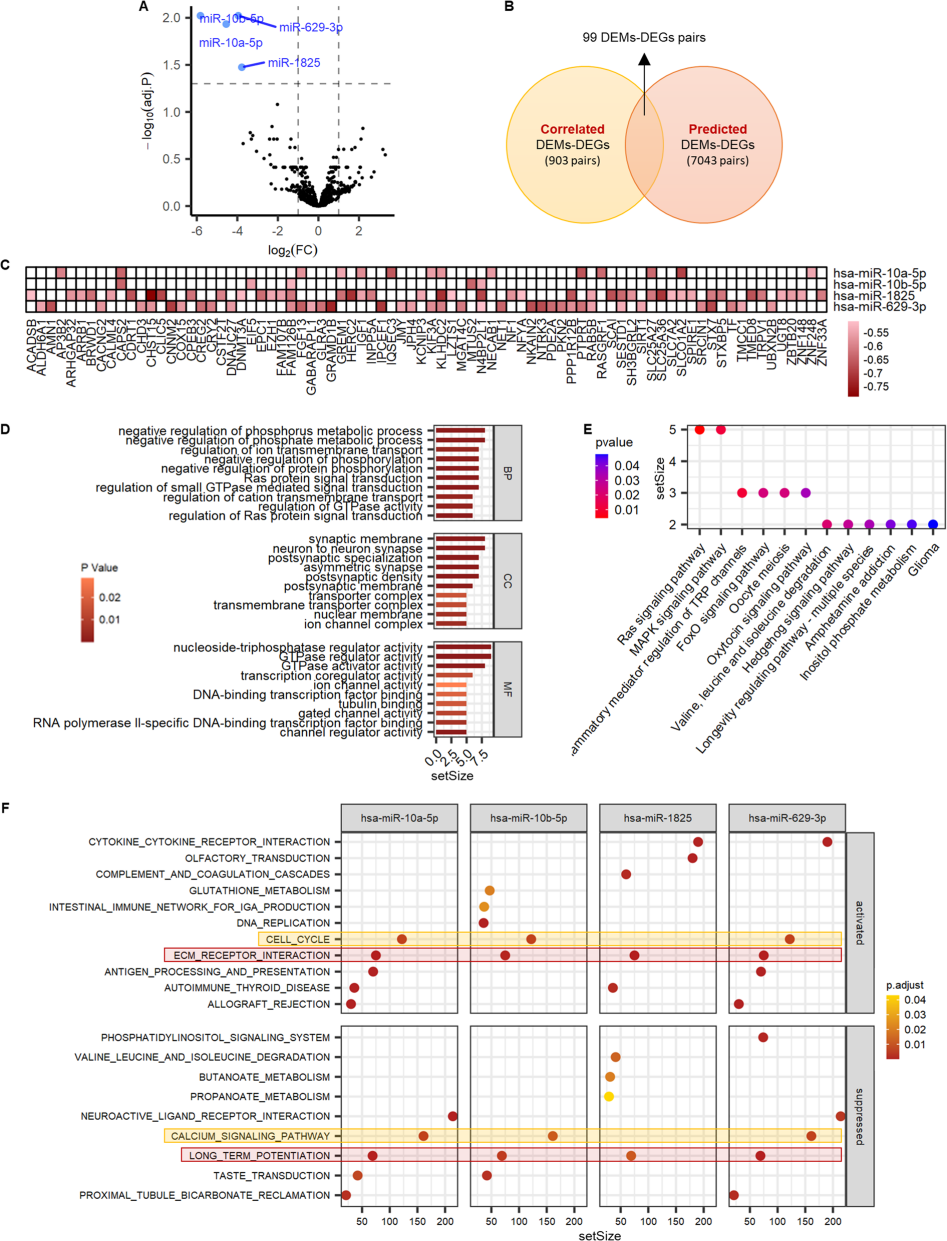
Identification and functional analysis of differentially expressed miRNAs (DEMs) in glioma-related epilepsy (GRE). **A** Volcano plots of DEMs. **B-C** Intersect of correlated DEMs-DEGs pairs and predicted DEMs-DEGs pairs. **D** Gene ontology (GO) annotations of DEM-related DEGs. **E** KEGG pathway enrichment for DEM-related DEGs analyzed by clusterProfiler package. **F** Gene set enrichment analysis (GSEA) terms were ordered according to their counts. FC, fold change; DEGs, differential expressed mRNAs; DEMs, differential expressed miRNAs; BP, biological process; CC, cellular component; MF, molecular function

There are 4 DEMs, hsa-miR-10a-5p, hsa-miR-10b-5p, hsa-miR-1825, and hsa-miR-629-3p, were discovered when miRNAs were screened for differential expression (Fig. 3A). Because miRNAs control gene expression by binding to their targets’ complementary sequences, the probable biological activities of DEMs can be deduced from the functions of their target genes. We found 903 pairs DEMs-DEGs with a high inverse association between miRNA and gene expression using Pearson’s correlation coefficients. MiRWalk (combining different algorithms, including TargetScan, miRDB, and miRTarBase) was used to find probable gene targets for each DEM. Following the intersection of the correlation and target prediction results, 99 DEMs-DEGs pairs were produced, which were then employed for functional analysis (Fig. 3B-C).

80 DEGs and 4 DEMs were found in the 99 DEMs-DEGs pairs. Figure 3D shows the enriched GO terms for these 80 DEGs. The most enriched GO annotations were negative regulation of phosphate metabolic process (biological process), synaptic membrane (cellular component), and nucleoside-triphosphatase regulator activity (molecular function). Notably, both Fig. 2B and Fig. 3D showed the enriched GO terms of nucleoside-triphosphatase regulator activity. The most enriched KEGG annotations of these 80 DEGs were the Ras signaling pathway and MAPK signaling pathway (Fig. 3E).

In addition, according to the strategy of “Guilt by association”, GSEA was used to predict the prospective roles of each important hub DEM (hsa-miR-10a-5p, hsa-miR-10b-5p, hsa-miR-1825, and hsa-miR-629-3p). We chose GSEA terms enriched by at least 3 miRNAs (≥ 75%) as the focus of further functional research. The results of Fig. 3F suggest that the function of hsa-miR-10a-5p, hsa-miR-10b-5p, and hsa-miR-629-3p may be related to activation of cell cycle and ECM receptor interaction, and suppression of calcium signaling pathway and long-term potentiation. Meanwhile, hsa-miR-1825’s function may be linked to activating ECM receptor interaction and suppressing long-term potentiation.

### Functional analysis of DELs in GRE

**Fig. 4:**
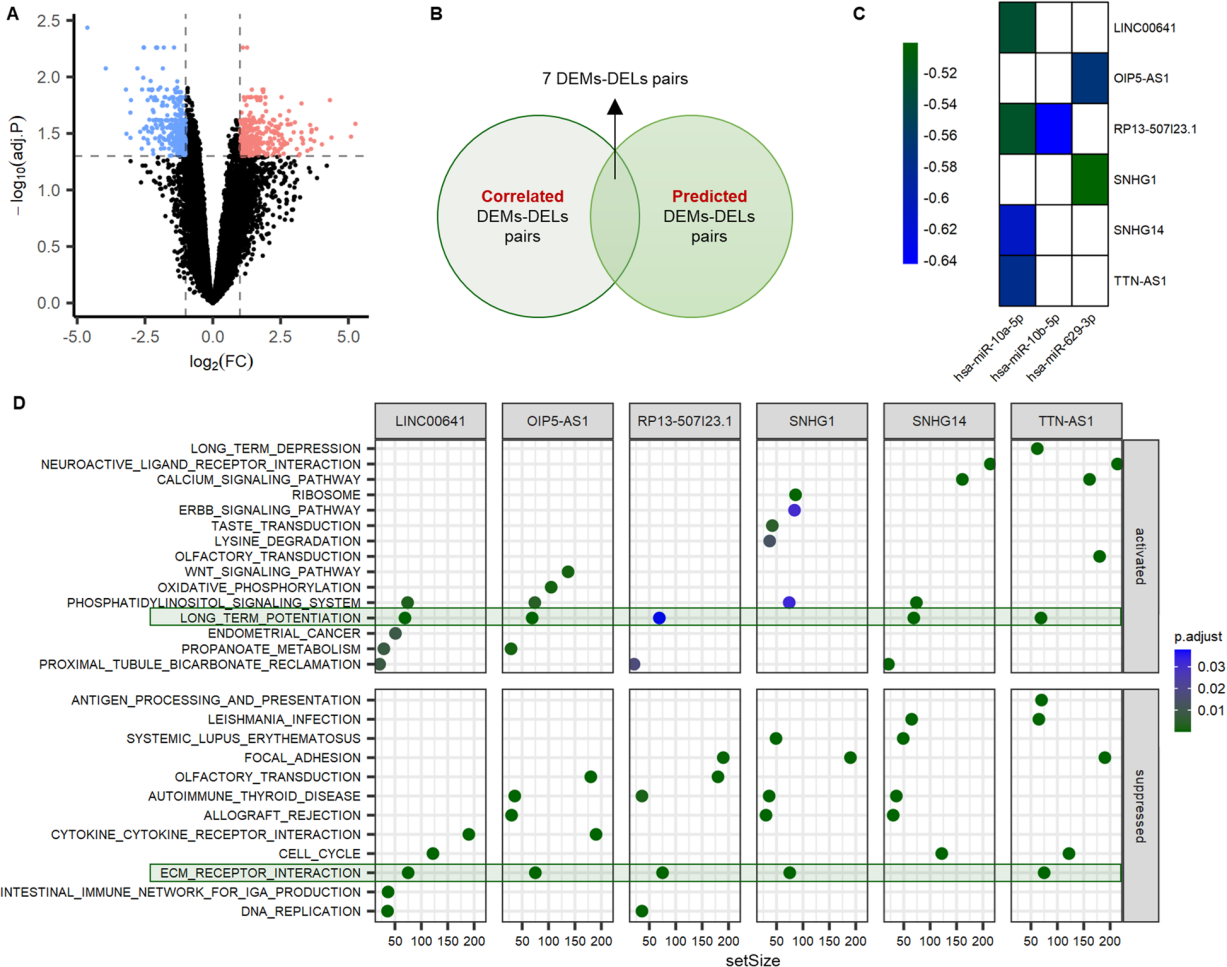
Identification and functional analysis of differentially expressed lncRNAs (DELs) in glioma-related epilepsy (GRE). **A** Volcano plots of DELs. **B-C** Intersect of correlated DEMs-DELs pairs and predicted DEMs-DELs pairs. **D** Gene set enrichment analysis (GSEA) terms were ordered according to their counts. FC, fold change; DEMs, differential expressed miRNAs; DELs, differential expressed lncRNAs

Between GRE and GNE, microarray-based research identified 37,581 lncRNAs and 669 DELs (324 up-regulated, 345 down-regulated) (Fig. 4A). Using Pearson’s correlation coefficients, we discovered 648 pairings DEMs-DEGs with a strong inverse relationship between miRNA and lncRNA expression. To explore candidate lncRNA targets for each DEM, ENCROI and LncBase 3.0 were employed. The intersection of the correlation and target prediction findings created 7 DEMs-DEGs pairings, which were subsequently used for functional analysis (Fig. 4B-C). In the 7 DEMs-DEGs couples, 3 DEMs (hsa-miR-10a-5p, hsa-miR-10b-5p, and hsa-miR-629-3p) and 6 DELs (LINC00641, OIP5-AS1, RP13-507I23.1, SNHG1, SNHG14, and TTN-AS1) were discovered. All these 6 DELs were up-regulated in GRE.

The 6 key hub DELs’ potential functions were predicted using GSEA. As the objective of the further functional study, we picked GSEA terms that were enriched by at least 5 DELs (≥ 75%). The role of LINC00641, OIP5-AS1, RP13-507I23.1, and TTN-AS1 may be associated with activation of long-term potentiation and repression of ECM receptor interaction, according to the results of Fig. 4D. Moreover, the functions of SNHG1 and SNHG14 may be connected to the repression of ECM receptor interaction and the activation of long-term potentiation, respectively.

### Construction of lncRNA-miRNA-Gene networks in GRE

**Fig. 5:**
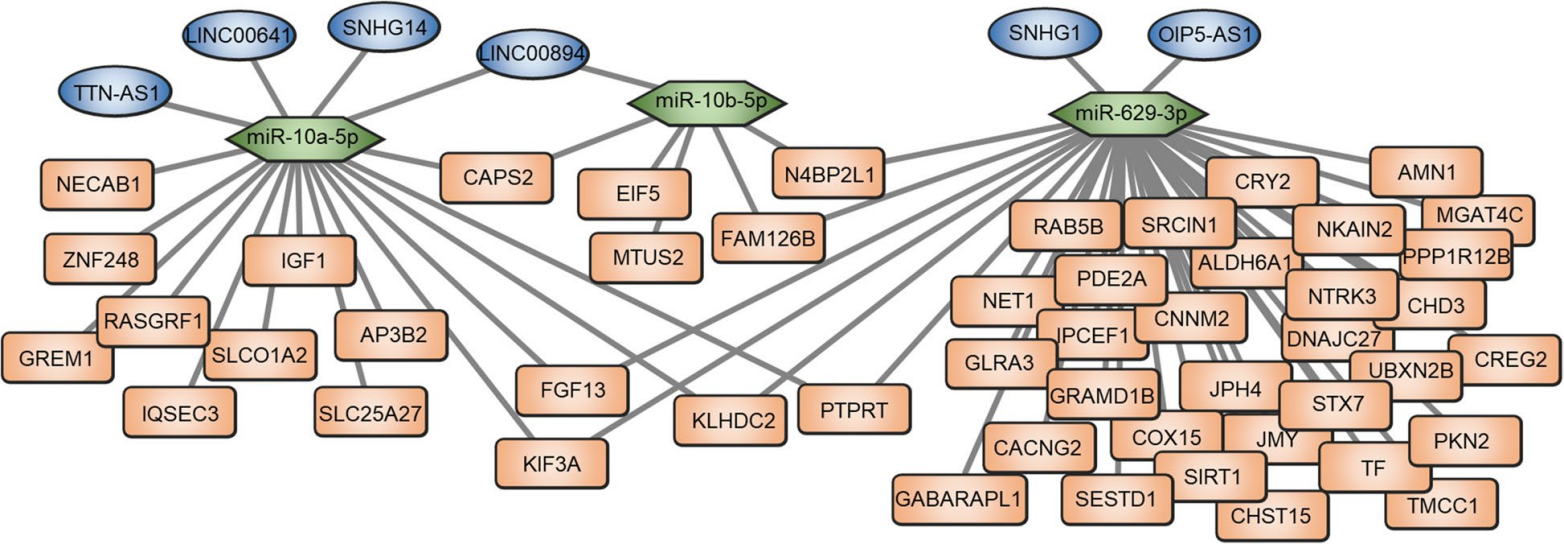
Construction of GRE-related lncRNA–miRNA–mRNA hypothesis network. GRE, glioma-related epilepsy

We constructed a lncRNA-miRNA-Gene interaction network via integrated analysis of DELs, DEMs, and DEGs to provide a complete understanding of GRE-regulated transcriptional processes (Fig. 5). There were 3 DEMs, 6 DELs, and 49 DEGs in the network.

**Fig. 6:**
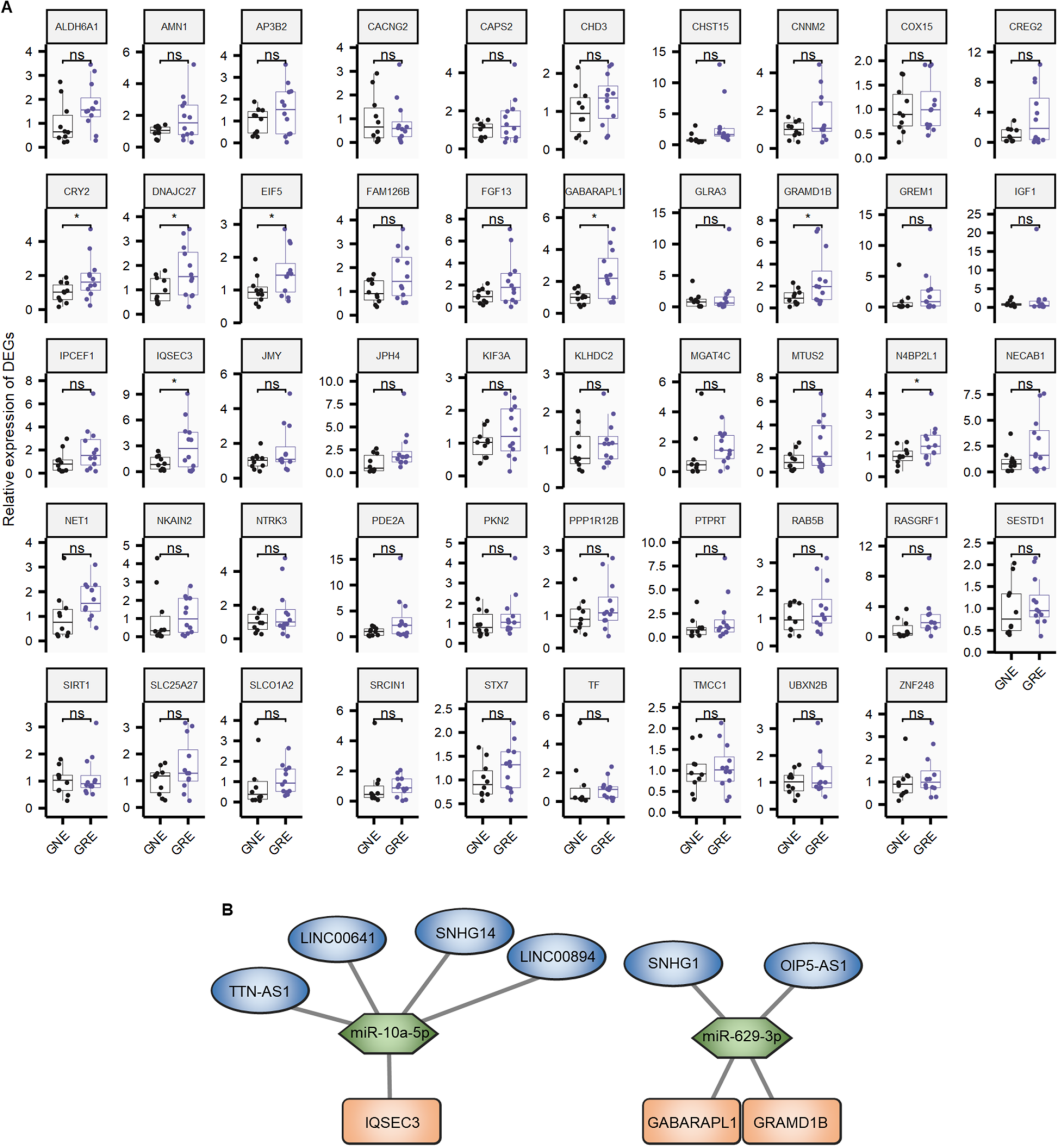
**A** Validation of the 49 DEGs expression in another cohort (10 GNE and 12 GRE). **B** GRE-related lncRNA–miRNA–mRNA hypothesis network associated with GABARAPL, GRAMD1B, and IQSEC3

To further enhance the persuasion of the research results and the value of the article, we carried out some experiments to verify the 49 DEGs. 10 GNE and 12 GRE samples were collected for PCR validation. The demographic characteristics of patients selected for validation was summarized in Table S1. As shown in Fig. 6A, The expression of GABARAPL1 (FC = 2.39, *p* = 0.011), GRAMD1B (FC = 2.72, *p* = 0.039), and IQSEC3 (FC = 3.03, *p* = 0.036) were more than twofold higher in the GRE group than in the GNE group in the validation cohort. On the other hand, the expression of CRY2 (FC = 1.86, *p* = 0.04), DNAJC27 (FC = 1.76, *p* = 0.043), EIF5 (FC = 1.54, *p* = 0.044), and N4BP2L1 (FC = 1.65, *p* = 0.041) did not exceed twofold. And the GRE-related lncRNA–miRNA–mRNA hypothesis network associated with GABARAPL1, GRAMD1B, and IQSEC3 was shown in Fig. 6B. In summary, we suggest that GABARAPL1, GRAMD1B, and IQSEC3 may underlie the pathogenesis of GRE.

## Discussion

Interventions with surgery, radiation, and chemotherapy in glioma patients has been shown to beneficial for seizure control, but GRE is commonly resistant despite regular anti-epileptic medications. The dynamic expression patterns of microRNA, mRNA, and lncRNA in brain tissues of patients with GRE or GNE were investigated in this work. 3 DEMs (miR-10a-5p, miR-10b-5p, miR-629-3p), 6 DELs (TTN-AS1, LINC00641, SNHG14, LINC00894, SNHG1, OIP5-AS1), and 49 DEGs were shown to be dysregulated in the development of GRE. The expression of GABARAPL1, GRAMD1B, and IQSEC3 were validated more than twofold higher in the GRE group than in the GNE group in the validation cohort. Pathways including ECM receptor interaction and long-term potentiation (LTP) may contribute to the disease’s progression.

The role of miR-10a-5p in the GRE or glioma has not been characterized so far. The role of these miRNAs in other cancers can provide some insight into their function. The expression of miR-10a-5p was dramatically reduced in breast cancer tissues and cell lines, and it was found to be adversely linked with MCF-7 cell growth [23]. Meanwhile, the tumor suppressor miR-10a-5p was down-regulated in oesophageal squamous cell carcinoma tissues and cancer cells and was linked to TNM stage and lymph node metastasis [24]. MiR-10b-5p is a microRNA that promotes tumor formation and is found on chromosome 2. In vitro and in vivo, miR-10b-5p significantly increased glioma migration and invasion [25]. In patients who received temozolomide chemotherapy, the level of miR-629-3p was shown to be substantially linked with overall survival [26].

TTN-AS1 and SNHG1 are lncRNAs that promote glioma development by upregulating RUNX1 [27, 28]. Tang et al. pointed out that TTN-AS1 induced malignant biological characteristics in glioblastoma cells via the miR-320b/EGR3/PKP2 axis [29]. By regulating miR-154-5p and miR-376b-3p, SNHG1 induces malignant biological characteristics in glioma cells [30]. In vivo, knockdown of OIP5-AS1 inhibited tumor growth, caused tumor regression, and extended survival [31, 32]. According to recent studies, LINC00641 inhibits glioma cell growth [33]. Overexpression of LINC00641 and SNHG14 inhibited cell growth but increased apoptosis in cells [34, 35].

In the absence of research reports on the above miRNAs or lncRNAs in GRE, the correlations between the signatures and glioma can only be viewed as a reference point. Further, it is essential to analyze the changes in expression profiles of the above signature-related mRNAs in the cohort of GRE to predict how the above signatures function.

First, it is worthy of concern that long-term potentiation and ECM receptor interaction pathways were enriched by all signatures (miR-10a-5p, miR-10b-5p, miR-629-3p, TTN-AS1, LINC00641, SNHG14, LINC00894, SNHG1, OIP5-AS1)-related genes. Figure 3F indicated that the function of the 3 down-regulated DEMs is related to the suppression of long-term potentiation and activation of ECM receptor interactions. Meanwhile, Fig. 4D suggested that the function of the 6 up-regulated DELs is related to activation of long-term potentiation and suppression of ECM receptor interactions.

LTP is commonly regarded as a possible cellular mechanism for learning and memory processes [36]. LTP has previously been reported to be reduced or eliminated after status epilepticus introduction [37]. In Fig. 2D, however, we observed that LTP was activated in GRE. All the 4 down-regulated DEMs were enriched in the LTP pathway in Fig. 3F. LTP activation and glutamate excitotoxicity can induce oligodendrocyte and neuronal death in Multiple Sclerosis patients [38]. The release of glutamic acid from the presynaptic neuron activates LTP by binding to an AMPAR on the postsynaptic neuron membrane as well as the NMDA receptor [39]. LTP in human tissue entails the activation of the NMDAR [40]. Excessive NMDAR activation is known to induce nerve cell dysfunction and death, leading to a variety of neurologic disorders [41]. After severe postsynaptic depolarization, NMDAR activation causes an increase in intracellular calcium ions, which is necessary for the biochemical process of LTP [42]. NMDAR mutations reduce the presence of glutamate, glycine, and Mg^2+^, resulting in NMDAR overactivation and neuronal hyper-excitability [41]. Interestingly, in our study, as an NMDAR, the gene GRIN1 was indeed up-regulated. A remarkable number of mutations in the NMDAR subunit have been discovered in seizure disorders producing diverse pediatric epilepsy syndromes, indicating that the NMDAR subunit may be a locus for epilepsy. NMDAR gain-of-function mutations may induce brain over-excitation, which might lead to epilepsy [43].

The extracellular matrix (ECM) of the brain should be regarded as a critical factor in the propagation of gliomas throughout the brain. Glioma cells move less when the ECM is less firm [44]. Patel et al. pointed out that the electrographic seizure-like events caused by the breakdown of the ECM increased the frequency of myoclonic seizures. These findings imply that an undamaged ECM inhibits the onset of seizures and that abnormalities in the ECM alone may be enough to cause at least some seizure activity [45]. This suggests that the inhibition of the ECM may promote the occurrence of epilepsy in gliomas.

Furthermore, Fig. 3F shows that the 3 down-regulated DEMs were likewise involved in calcium signaling pathway suppression. Moreover, in Fig. 2D, the calcium signaling pathway was activated in the GSEA result for all mRNAs in GRE. Calcium plays an important role in neurotransmission, exocytosis, and intracellular signaling. Calcium channel activation may promote glioma transformation and development by increasing metabolic switching, glial proliferation, angiogenesis, invasion, and migration [46]. Calcium signaling has become widely acknowledged as a significant element in epileptogenesis during the last decade. These include immediate impacts on membrane excitability caused by calcium influx and delayed processes mediated by G-protein coupled pathways [47]. As a result, down-regulation of miR-10a-5p, miR-10b-5p, and miR-629-3p may contribute to calcium signaling activation.

Taken together, the expression of 3 DEMs (miR-10a-5p, miR-10b-5p, miR-629-3p), 6 DELs (TTN-AS1, LINC00641, SNHG14, LINC00894, SNHG1, OIP5-AS1), and 49 DEGs have substantial links to GRE, according to our findings. Pathways including ECM receptor interaction and long-term potentiation may contribute to the disease’s progression. These findings highlight the value of molecular GRE profiling, which has crucial implications for GRE management.

The expression of GABARAPL1, GRAMD1B, and IQSEC3 were validated more than twofold higher in the GRE group in the validation cohort. The gene GABARAPL1 (GABA Type A Receptor Associated Protein Like 1) codes for a protein. Microtubule binding and GABA receptor binding are two Gene Ontology (GO) annotations for this gene. GABA and glutamate are the two major neurotransmitters that influence seizures, and the balance of the two neurotransmitters in the nervous system is particularly important for the prevention of epileptogenesis [48]. In another study on GRE, the authors found a disturbance in the glutamatergic neurotransmitter pathway [49]. Notably, CELF4, SLC17A7, CAMK2A, CXCL8, H19 and VEGFA were found to be significantly differentially expressed in GRE versus GNE in this study, whereas these genes were not found to be significantly differentially expressed in our study. Perhaps, differences in results may be due to ethnic differences, tumour characteristics or small sample sizes. The GRAMD1B gene is thought to be involved in cholesterol binding, cholesterol transfer, and phospholipid binding. There are no reports of GRAMD1B being associated with epilepsy or glioma. IQSEC3 is thought to function as a guanyl-nucleotide exchange factor. In the Hippocampus, IQSEC3 loss disrupts GABAergic Synapse Maintenance and lowers Somatostatin Expression [50]. In mice, reducing activated microglia reduces seizure progression triggered by IQSEC3 loss [51]. We speculate that the elevated IQSEC3 in GRE may be a stressful compensatory effect.

Using an integrated approach that includes lncRNAs, miRNAs, and mRNAs, we can screen for possible genetic and epigenetic biomarkers in GRE at the transcriptional level and elucidate their regulatory functions, which will be critical for identifying diagnostic biomarkers and therapeutic targets for GRE. Extending the current findings to the diagnosis and treatment of GRE in people, however, remains a significant hurdle. In any case, the discovery of these genetic and epigenetic indicators advances the development of GRE diagnostic and therapy options.

There are several drawbacks to this study. Although the microarrays’ high sensitivity and specificity give a high degree of confidence in the current findings. The limited sample sizes make it impossible to rule out the possibility of false negatives. Consequently, these findings must be empirically validated in future investigations. In the fields of GRE research, our findings provide a valuable list of study targets. In this study, we cannot guarantee that 100% of the tissue samples taken were glioma cells and did not contain any paraneoplastic cells. Therefore, we suggest that other investigators use single-cell sequencing in their next studies to assess the difference in the role of cancer cells versus paraneoplastic cells in epileptogenesis. In addition, in vivo and in vitro functional validation, relying on disease models, could also be the next step in assessing the role of these genes in the etiology of GRE.

## Supplementary Information

**Additional file 1: Table S1. **Clinical data of glioma patients with or without epilepsy for validation.

## Data Availability

All Microarray raw data have been deposited in the Gene Expression Omnibus (GEO) under accession GSE199759 (https://www.ncbi.nlm.nih.gov/geo/query/acc.cgi?acc=GSE199759). Please contact Zhao-Qian Liu (zqliu@csu.edu.cn) if someone wants to request the data from this study.

## Abbreviations

DEGs: Differentially Expressed mRNAs
DELs: Differentially Expressed lncRNAs
DEMs: Differentially Expressed miRNAs
ECM: Extracellular matrix
FC: Fold change
GABA: γ-Aminobutyric acid
GNE: Glioma without epilepsy
GO: Gene Ontology
GRE: Glioma-related epilepsy
GSEA: Gene Set Enrichment Analysis
KEGG: Kyoto Encyclopedia of Genes and Genomes
LTP: Long-term potentiation
GEO: Gene Expression Omnibus
