# Supplementary material for: Integrative analysis of expression profile indicates the ECM receptor and LTP dysfunction in the glioma-related epilepsy
Source: BMC Genomics. 2022 Jun 8;23:430. doi: 10.1186/s12864-022-08665-8 (PMC9175475; doi:10.1186/s12864-022-08665-8)
Supplement: Supplementary file 1 — Additional file 1: Table S1. Clinical data of glioma patients with or without epilepsy for validation. [file 12864_2022_8665_MOESM1_ESM.docx]

**Table S1** | Clinical data of glioma patients with or without epilepsy for validation

| **Characteristic** | **Classes** | **GNE^a^** | **GRE^b^** |
| --- | --- | --- | --- |
| Gender | Male | 6 | 8 |
|  | Female | 4 | 4 |
| Age | Age≤50 | 5 | 11 |
|  | Age＞50 | 5 | 1 |
| Epilepsy | YES | 0 | 12 |
|  | NO | 10 | 0 |
| Tumor grade | Ⅰ-Ⅲ | 6 | 12 |
|  | Ⅳ | 4 | 0 |
| Site | Temporal lobe | 4 | 5 |
|  | Frontal lobe | 6 | 5 |
|  | Parietal lobe | 0 | 2 |
|  | Occipital lobe | 0 | 0 |
| IDH1 | Wide type | 8 | 10 |
|  | Mutant type | 2 | 2 |

*^a^ glioma without epilepsy.*

*^b^ glioma-related epilepsy.*

*All gliomas were primary without radiotherapy or chemotherapy was received before surgery. The type of epilepsy in GRE was sustained epilepsy.*
